# Development and Internal Validation of a Multivariable Prediction Model for Mortality After Hip Fracture with Machine Learning Techniques

**DOI:** 10.1007/s00223-024-01208-1

**Published:** 2024-04-16

**Authors:** Mathias Mosfeldt, Henrik Løvendahl Jørgensen, Jes Bruun Lauritzen, Karl-Åke Jansson

**Affiliations:** 1 Department of Orthopaedics, Karolinska University Hospital, Stockholm, Sweden; 2 Department of Molecular Medicine and Surgery, Karolinska Institutet, Stockholm, Sweden; 3 Department of Clinical Biochemistry, Hvidovre Hospital, Hvidovre, Denmark; 4 Department of Clinical Medicine, University of Copenhagen, Copenhagen, Denmark; 5 Department of Orthopaedic Surgery, Bispebjerg Hospital, University of Copenhagen, Copenhagen, Denmark; 6 Department of Orthopaedics, Södersjukhuset, Stockholm, Sweden

**Keywords:** Hip fracture, Mortality, Machine learning, Prediction, Random forest

## Abstract

In order to estimate the likelihood of 1, 3, 6 and 12 month mortality in patients with hip fractures, we applied a variety of machine learning methods using readily available, preoperative data. We used prospectively collected data from a single university hospital in Copenhagen, Denmark for consecutive patients with hip fractures, aged 60 years and older, treated between September 2008 to September 2010 (*n* = 1186). Preoperative biochemical and anamnestic data were used as predictors and outcome was survival at 1, 3, 6 and 12 months after the fracture. After feature selection for each timepoint a stratified split was done (70/30) before training and validating Random Forest models, extreme gradient boosting (XGB) and Generalized Linear Models. We evaluated and compared each model using receiver operator characteristic (ROC), calibration slope and intercept, Spiegelhalter’s z- test and Decision Curve Analysis. Using combinations of between 10 and 13 anamnestic and biochemical parameters we were able to successfully estimate the likelihood of mortality with an area under the curve on ROC curves of 0.79, 0.80, 0.79 and 0.81 for 1, 3, 6 and 12 month, respectively. The XGB was the overall best calibrated and most promising model. The XGB model most successfully estimated the likelihood of mortality postoperatively. An easy-to-use model could be helpful in perioperative decisions concerning level of care, focused research and information to patients. External validation is necessary before widespread use and is currently underway, an online tool has been developed for educational/experimental purposes (https://hipfx.shinyapps.io/hipfx/).

## Introduction

Hip fractures are one of the most common orthopedic injuries that require hospitalization and has vast implications for patients and healthcare providers alike. Mortality has been shown to be 5–8-fold higher during the first three months after surgery for hip fracture [1] and incidence rates has been estimated to 2.7 million patients worldwide [2]. Costs from hip fractures in the European Union alone has been estimated to be 19,000 million € annually [3].

Estimating the likelihood of mortality after this widespread and costly injury could be useful in many settings. Perhaps most importantly as an aid when providing patients and their relatives with insight as to the severity of the injury, but also for caregivers to identify patients with a higher risk that might benefit from an elevated level of care such as more intensive monitoring, specialized orthogeriatric care or to triage patients for expedited surgery. Decisions regarding choice of implant for neck of femur fractures that take life expectancy into consideration has also been discussed to avoid overtreating very frail patients with a prosthesis and the added surgical stress and prolonged rehabilitation that comes along with this procedure compared to percutaneous screws. Furthermore, on a larger scale, research on methods to decrease postoperative mortality in this heterogenic group of patients could benefit from estimations of mortality to focus efforts where they are most needed. From a public health perspective, this type of estimations could be useful in comparisons between institutions to adjust for case-mix.

There have been several publications of systems for estimating mortality after hip fracture [4–10], however all the methods listed above employ traditional frequentist statistics. A wide range of studies has been published demonstrating benefits and excellent performance using machine learning techniques (ML) for prediction modeling in orthopedics and other fields of medicine [11, 12]. Some more recent studies have sought to explore prediction of mortality after hip fracture with ML and achieved good results. However, these studies either use a large number of pre- and postoperative parameters making them impractical for assessment of patients upon admission to the hospital [13, 14], are of a descriptive nature concerning development of models that are not accessible to test or validate on other populations [14–16] or focus on a subgroup such as neck of femur fractures, patients that are critically ill or identifying patients with very high short term mortality risk after surgery [17–19].

We sought to develop an accurate prediction model for mortality at 1, 3, 6 and 12 months after hip fracture with ML techniques using only parameters available at the time of admission.

Furthermore, we wanted to create a freely available online tool so that estimations could be used to aid in clinical decisions.

## Methods

### Source of Data

The study is based on a database from Bispebjerg University Hospital in Copenhagen, Denmark, that consists of 1601 hip fracture patients with 65 recorded variables for each patient. Blood samples were taken on admission and data were recorded by the attending physician or a study nurse for the purpose of the database. All hip fracture patients with no known malignant disease were included from September 2008 to September 2010.

### Participants

The database consisted of 1465 patients over 60 years of age that had suffered a hip fracture. Patients below the age of 60 years were excluded as mortality rates in younger patients that suffer hip fractures are drastically lower and we wanted to focus on potentially frail patients with health issues related to aging [20, 21]. Patients with ASA 5 or 6 (*n* = 1) were excluded. All patients were treated according to local guidelines incorporating a fast track program [22]. During the study period a change was made in the perioperative care of the patients while in the hospital as a dedicated orthogeriatric ward was introduced with both geriatricians and orthopedics attending to patients [23]. This was entered into the dataset as a potential variable for survival. All data analysis was done on completely anonymized datasets.

### Outcome

Follow-up data on mortality was collected from the Danish civil registration system on the 10th of October 2013, so records existed for at least 36 months of follow-up time for the patients included last. All citizens and anyone residing legally in Denmark are registered in the Danish Civil Registration System using a unique 10-digit civil registration and vital status was available for all patients in the study.

### Predictors

We considered the following variables in the registry for inclusion in the models. They included: age, sex, types of medication at admission, orthogeriatrics (y/n), anesthesia type, fracture type, type of operation, type of permanent residence (own home, nursing home, homeless), where patients were admitted from (own home, assisted living, rehab, hospice, hospital, nursing home), new mobility score (NMS) [24], American Society of Anesthesiologists physical status (ASA) score, body mass index (BMI), survival (yes/no) at 1, 3, 6 and 12 months after admission, biochemistry (hemoglobin, potassium, sodium, creatinine, calcium, albumin, glucose).

### Missing Data and Feature Selection

One hundred and nine patients had no registered blood samples and were excluded from further calculations. Blood samples as well as all other data concerning patients were collected and recorded prospectively for the purpose of the database at the time of admission and there was no later changes to this data except for the inclusion of vital status. At the time of calculations, the data were anonymous and there was way to retrospectively use the personal identification numbers to retrieve data from the hospital charts while respecting the boundaries of the ethical permit and patient confidentiality.

Of the 1356 patients that remained, 169 patients were missing both albumin and calcium. There was no statistical significance between mortality and the cases with completely missing blood samples or the group that was missing calcium and albumin using the chi-squared test when compared to rest of the data. As such they were assumed to be missing independently of the outcome and listwise deletion should be unbiased. This was considered a better option than imputing a relatively large proportion of data for these parameters as the decreased amount of data were considered unlikely to have a significant effect on development of prediction models. Of the 1186 patients that remained most parameters had complete data and the parameters that had missing values had less than 10% missing and this was considered an acceptable amount for imputation.

Imputation was done using a random Forest imputation algorithm for missing data [25] that has been shown to outperform several of the other commonly used methods such as KnnImpute, and multiple imputation by chained equations (MICE).

The data were split in training/test partitions with a 70/30 stratified split to ensure that similar proportions of the outcome are preserved in each set.

The Boruta algorithm [26] was used for selecting parameters of importance for the different timepoints (Table 2) on the training set. This is a feature selection algorithm that works by comparing the parameters importance in relevance to the outcome with the importance of the same values permuted at random. It returns all relevant features in relation to the outcome for building prediction models. Some feature engineering was done after noting that digoxin and vitamin K-antagonists were selected by Boruta for several timepoints. Several combinations of having cardiac medications, antihypertensive and anticoagulants in the medical history were created and evaluated for inclusion by repeating the Boruta algorithm. Having prescribed diuretics, betablockers, digoxin, vitamin k antagonists and organic nitrates alone or in combination and was used as a feature and had a higher importance in combination than as individual parameters.

Blood sample values were converted to categories of normal or low for albumin and hemoglobin, normal or high for creatinine, and finally low, normal or high for potassium and calcium. While it might be beneficial to differentiate between extreme variations of abnormal it was determined nonsensical to treat these values as continuous as there should be no difference in variations within the normal range.

Finally, the same parameters and partitions for each timepoint were used to train a random forest (RF), an extreme gradient boosting (XGB) and a genralized linear model (GLM). According to Breiman and Cutler who created the Random Forest algorithm, there is no need for a separate test set or cross-validation when developing models using this technique as the algorithm effectively performs internal leave one out cross-validation (LOOCV) during training [27]. Training was done with LOOCV on the training set for the GLM and XGBoost models to make results comparable to the RF model. Hyperparameter tuning was performed using a gridsearch and internal tenfold cross-validation on the training sets for the RF and the XGBoost models and dummy encoding of categorical variables was done for the XGBoost models.

The final models were used to make predictions on the hold out test sets, performance was assessed by the area under the curve (AUC) of receiver operating characteristics (ROC) curves. Calibration slope and intercept, Spiegelhalter’s z-test and Decision Curve analysis (DCA) were assessed for all models. R software was used for calculations [28]. The manuscript was prepared according to the TRIPOD statement [29].

## Results

**Fig.1:**
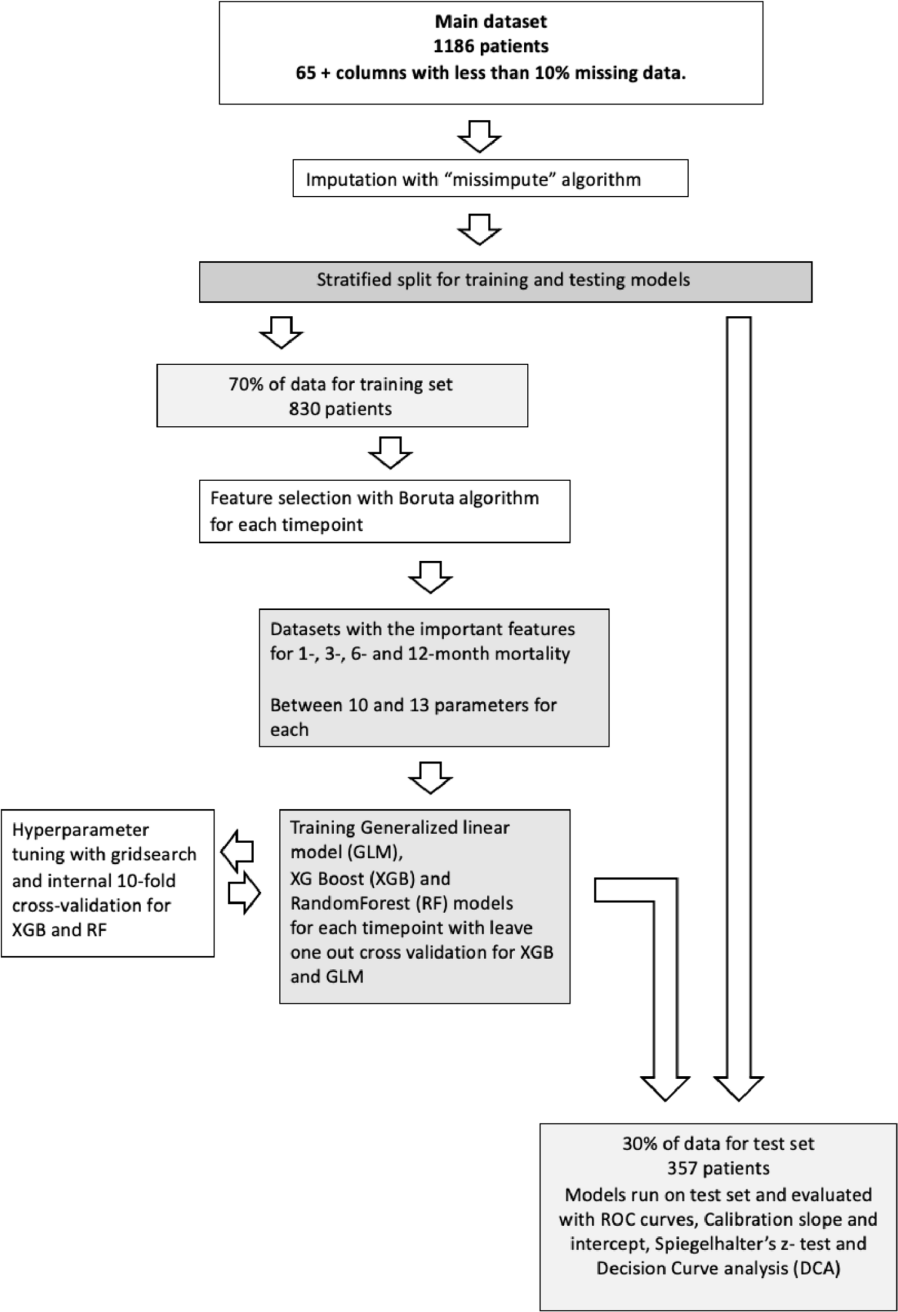
Flowchart for building models

Using only parameters available at admission we were able to train ML models to estimate for 1, 3, 6 and 12 month mortality after hip fracture with good to excellent discrimination on ROC curves. The parameters chosen by the Boruta algorithm for the different timepoints where slightly different but “Permanent/ registered residence”, “Admitted from”, “New Mobility Score”, ASA, potassium, creatinine, albumin, “cardiac medications y/n” and age were relevant for all timepoints. The models performed similarly with an AUC close to 0.80 for all timepoints (Fig. 1).

The RF models had marginally better discrimination than the other models with AUC values of 0.79 (CI 0.72–0.85), 0.80 (CI 0.74–0.85), 0.79 (CI 0.74–0.84), 0.81 (CI 0.76–0.85) for 1, 3, 6 and 12 month mortality, respectively.

**Fig. 2:**
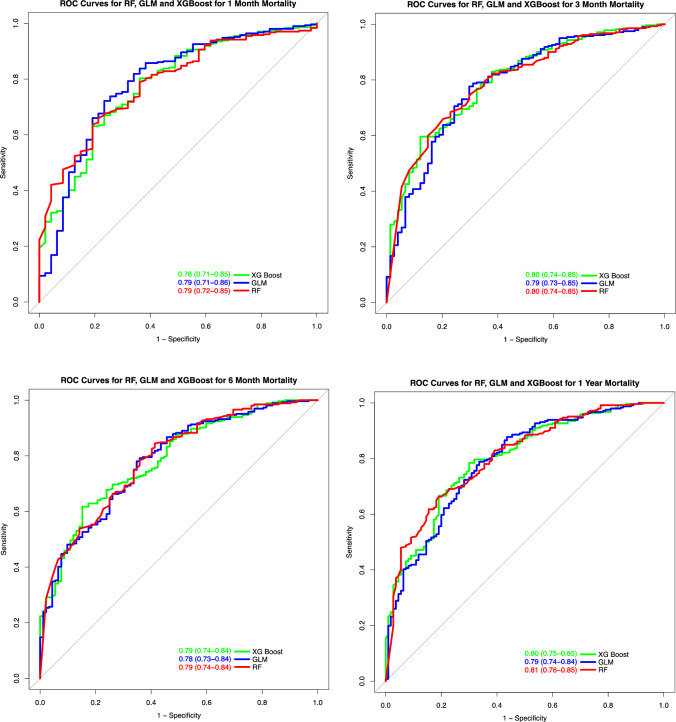
ROC curves for 1, 3, 6 and 12 month mortality

ROC curves and AUC values with CI for all timepoints and models are presented in Fig. 2.

While models performed in a similar manner for discrimination, there was big differences in calibration. For datasets of this size, flexible calibration plots will be unstable so mean calibration, calibration slope and intercept are reported as recommended by van Calster et al. [30].

The mean calibration is the average predicted risk compared to the overall event rate for the outcome predicted. The RF model consistently underestimated quite severely while the XGB and GLM models were well aligned with the overall event rate for all timepoints.

The calibration slope has a target value of 1 and is used to evaluate if estimations are exaggerated or too extreme (< 1) or too conservative (> 1) and has also been referred to as the “spread” of the estimates. The intercept has a target value of 0 and is a measure of over- (< 0) or under-estimation (> 0) and should be read together with the slope and indicates calibration across a range of estimations. The XGB model was fairly well calibrated for all timepoints, the GLM uncalibrated for 1 month mortality and all of the RF models were poorly calibrated.

Finally, Spiegelhalter’s *z*-test was also used as a measure of calibration. A set of observations and associated probabilities are used, and the null hypothesis of the statistical test is that models are well calibrated. *P* values that are statistically significant indicate poor calibration and the degree of miscalibration corresponds to larger absolute values of *z* regardless of whether values are positive or negative (Table 1).

**Table 1:** Abbreviated (abbreviated, all data included in Appendix)

Patients after exclusion criteria, *n* = 1186		
Mortality 1 month (% of total)	145 (12.2)	
Mortality 3 months (% of total)	242 (20.4)	
Mortality 6 month (% of total)	308 (25.9)	
Mortality 1 year (% of total)	378 (31.8)	

Patient characteristics stratified by 1 year survival				
	No	Yes	*p*-test	NA
Survival 1 year (% of total)	378 (31,9)	808 (68,1)		
Age (mean (SD))	86.10 (8.38)	81.33 (9.24)	< 0.001	–
Sex = male (%)	104 (27.5)	202 (25.0)	0.395	–
BMI (mean (SD))	22.05 (3.47)	22.88 (4.15)	0.001	42
Creatinin, μmol/L (mean (SD))	103.84 (74.09)	77.71 (36.54)	< 0.001	1
Hemoglobin, mmol/L (mean (SD))	7.36 (1.03)	7.74 (1.05)	< 0.001	–
Potassium, mmol/L (mean (SD))	4.02 (0.60)	3.86 (0.48)	< 0.001	3
Sodium, mmol/L (mean (SD))	137.70 (4.72)	137.68 (4.44)	0.950	–
Calcium, mmol/L (mean (SD))	2.26 (0.17)	2.26 (0.13)	0.685	37
Albumin, g/L (mean (SD))	36.49 (5.30)	38.69 (4.27)	< 0.001	11
Glucose, mmol/L (mean (SD))	6.80 (1.99)	6.64 (2.12)	0.228	22
Admitted from (%)			< 0.001	
Assisted living	18 (4.8)	52 (6.4)		
Hospice	2 (0.5)	0 (0.0)		
Hospital	7 (1.9)	11 (1.4)		
Nursing home	155 (41.0)	126 (15.6)		
Own home	180 (47.6)	603 (74.6)		
Rehab	16 (4.2)	16 (2.0)		
Permanent/ registered residence (%)			< 0.001	7
Homeless	0 (0.0)	1 (0.1)		
Nursing home	154 (40.7)	129 (16.0)		
Own home	220 (58.2)	675 (83.5)		
New mobility score total (%)			< 0.001	96
0	15 (4.0)	10 (1.2)		
1	8 (2.1)	6 (0.7)		
2	60 (15.9)	67 (8.3)		
3	29 (7.7)	49 (6.1)		
4	60 (15.9)	83 (10.3)		
5	12 (3.2)	46 (5.7)		
6	54 (14.3)	116 (14.4)		
7	11 (2.9)	50 (6.2)		
8	1 (0.3)	15 (1.9)		
9	83 (22.0)	315 (39.0)		
ASA classification (%)			< 0.001	47
1	7 (1.9)	68 (8.4)		
2	134 (35.4)	435 (53.8)		
3	194 (51.3)	266 (32.9)		
4	20 (5.3)	15 (1.9)		
Cardiac medication = YES (%)	236 (62.4)	358 (44.3)	< 0.001	

*P*-test was calculated with chi- square test for categorical variables (with continuity correction) and oneway test for continuous variables (with equal variance assumption, i.e., regular ANOVA)

The XGB models were the best calibrated overall, the GLM had significant *P* values for Spiegelhalter’s *z*-test for the 1- and 3 month models indicating that these were not well calibrated, and the RF was very poorly calibrated on all measurements and timepoints. *P*-values were significant for all RF models and the *z*-test had very high values in line with the mean calibration and the values of the calibration slope that also indicated poor calibration of these models.

All calibration measures are reported in Table 2.

**Table 2:** Table of results

Prediction of mortality after hip fracture	1 month		3 months		6 months		1 year	
Overall event rate in test set	0.13		0.19		0.26		0.31	
Calibration measures	Average predicted risk	Slope, intercept and Spiegelhalter *z* test	Average predicted risk	Slope, intercept and Spiegelhalter *z* test	Average predicted risk	Slope, intercept and Spiegelhalter *z* test	Average predicted risk	Slope, intercept and Spiegelhalter *z* test
XGBoost	0.11	S: 0.99 I: 0.22 S(z): 1.53 S(p): 0.12	0.21	S: 0.83 I: − 0.03 S(z): 0.11 S(p): 0.90	0.25	S: 1.12 I: 0.20 S(z): 0.02 S(p): 0.99	0.30	S: 1.20 I: 0.19 S(z): − 0.77 S(p): 0.44
Generalized linear model	0.10	S: 0.31 I: − 1.02 S(z): 2.1 S(p): 0.03	0.19	S: 0.99 I: 0.22 S(z): 1.75 S(p): 0.08	0.25	S: 0.88 I: − 0.01 S(z): 1.22 S(p): 0.22	0.30	S: 0.82 I: − 0.08 S(z): 0.08 S(p): 0.94
Random forest	0.09	S: 0.60 I: − 0.18 S(z): 4.5 S(p): 0.00	0.04	S: 0.49 I: 0.79 S(z): 18.71 S(p): 0.00	0.07	S: 0.60 I: 1.00 S(z): 16.93 S(p): 0.00	0.14	S: 0.81 I: 1.03 S(z): 11.20 S(p): 0.00
Parameters in prediction models								
Where was patients admitted from	√		√		√		√	
Permanent residence status	√		√		√		√	
New mobility score	√		√		√		√	
ASA	√		√		√		√	
BMI					√		√	
Age	√		√		√		√	
Sex	√		√		√		√	
Treated with cardiac medication	√		√		√		√	
Albumin	√		√		√		√	
Hemoglobin					√		√	
Creatinine	√		√		√		√	
Calcium			√		√		√	
Potassium	√		√		√		√	

The RF models also performed notably worse on DCA plots for all timepoints than the other models. The DCA calculates “net benefit” of using a model to choose patients for a treatment compared to treating all or none, or compared to using another model for this purpose. Considering the DCA for 3 month mortality, a “net benefit” of 0.10 at the 20% probability threshold for the outcome could be interpreted as identifying 10 true positives when using the model on a population of 100 patients.

**Fig. 3:**
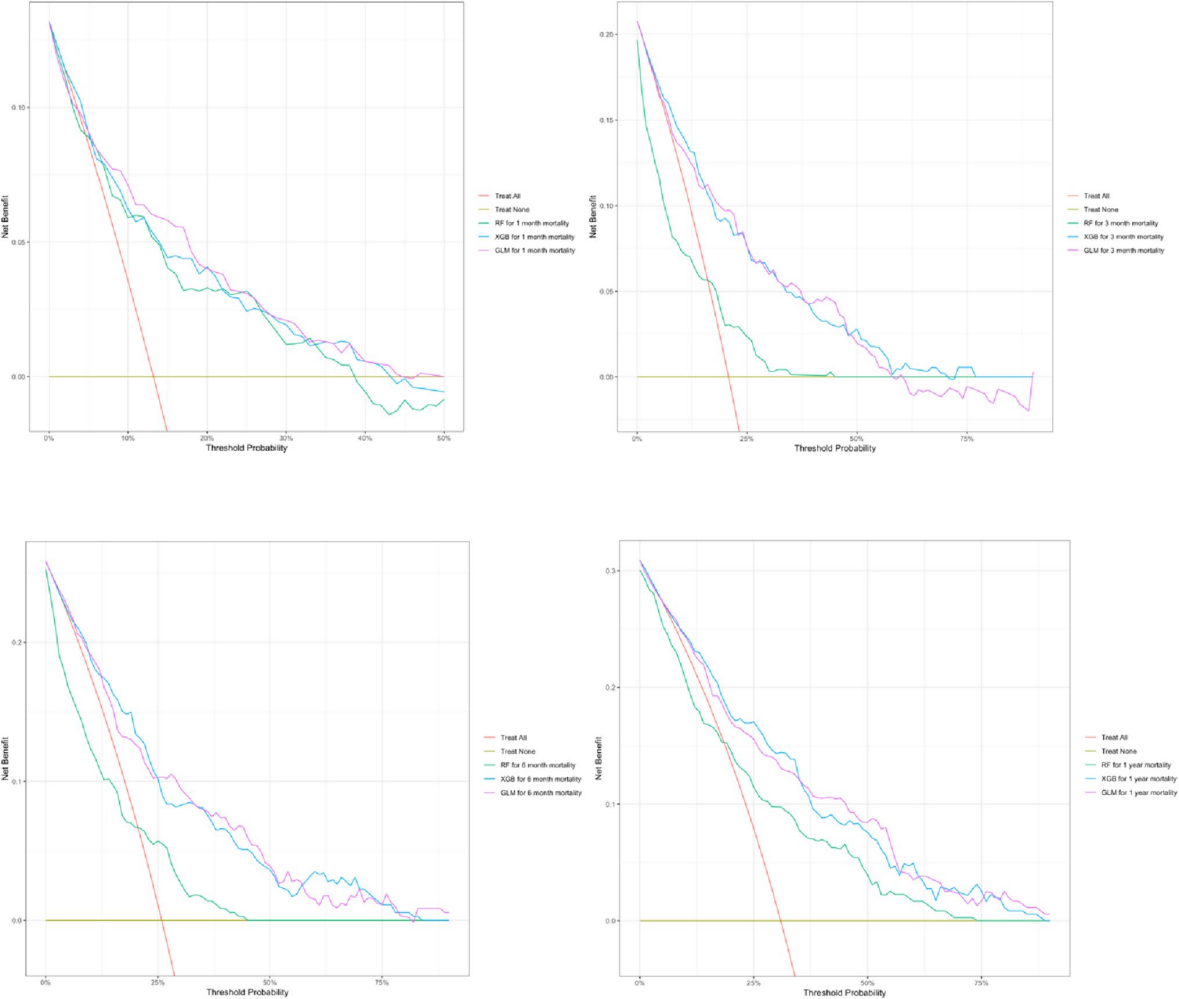
DCA curves for 1-, 3-, 6- and 12 month mortality

As the models are intended to be used in a wide variety of settings, one of which is providing information to patients and their relatives, the thresholds are set so that the entire spectrum of positive values are shown. In most settings for clinical use, it is probably thresholds in the lower end that is interesting as the harm of false positives and “unnecessary treatment” is expected to be low with interventions such as increased monitoring or expedited surgery. Of course, if models are used to restrict treatment that could otherwise be beneficial this must be taken into account, however the GLM and the XGB models had a higher “net benefit” for all timepoints than the RF model, indicating that they are better suited for all clinical decisions across the range of thresholds demonstrated. The DCA curves for all models and timepoints are available in Fig. 3, for the 1 month model the DCA is truncated at the threshold of 0.5 as all models had negative values after this point.

The XGB model had the overall best performance, so using the “Shiny” software package in RStudio an interactive app was built for educational purposes to explore how the models could function in clinical practice. The “Shiny” package provides a coding language to create stand-alone web applications that can execute models developed in RStudio based on the input provided in the app. External validation of the models on other patient populations is underway and should be reviewed before putting models to widespread clinical use. The application is available online for evaluation (https://hipfx.shinyapps.io/hipfx/).

## Discussion

This study demonstrated the utility of machine learning techniques in estimating the likelihood of mortality in hip fracture patients. The developed models achieved acceptable to excellent results for both GLM, XGB and Random Forest modeling determined by AUC on ROC curves but with acceptable calibration only for the XGB model. The RF models seem much less suited for clinical use as they were poorly calibrated and had less net benefit on DCA curves. The GLM and the XGB was fairly similar for 6- and 12 month mortality and had a DCA indicating that they could be useful clinically for all timepoints. Overall, the XGB model was the most promising and was well calibrated for all timepoints with non-significant values on Spiegelhalter’s *z*-test.

These models will need to be externally validated on a different patient material to further corroborate the results.

The Notthingham hip fracture score (NHFS) seems to be most popular of the previously published models for prediction of mortality after hip fracture. This scoring system incorporates age, sex, number of co-morbidities, mini-mental test score at admission, hemoglobin level at admission, presence of malignant disease and whether patients were living in an institution at the time of the fracture as factors. It has been externally validated several times [31–35], with results varying from an AUC of 0.67 in a Swedish study by Jonsson et al. to 0.83 in a smaller Greek study by Tilkeridis et al. This might reflect how different parameters have varying importance in different populations, furthermore how predicting future outcomes is not an exact science.

Several of the previous articles that utilize ML techniques to model mortality after hip fracture have used a mixture of pre- and postoperative parameters as predictors in the final models so they are not directly comparable.

A large study of 19,835 US hip fracture patients identified 47 important parameters from a database with 150 available parameters of mixed pre, intra and postoperative data and developed artificial neural network (ANN), logistic regression and naïve Bayes models with excellent results for 1 month mortality with 0.92, 0.87 and 0.83, respectively. It is unclear what the timeframe was concerning follow-up of postoperative parameters. Feature selection was done by backward variable selection and several of the parameters in our study also had importance in this study such as BMI, creatinine, hypoalbuminemia, pre-op mobility aid, age and sex [13].

Similarly, a different study from the US on 17,140 patients included length of hospital stay as a predictor as well as sociodemographic and clinical factors to predict 30 day and 1 year mortality after hip fracture using logistic regression and multilayer perceptron modeling and obtained an AUC of around 0.76 for both models and timepoints [15]. No holdout or test set was used, the performance was measured as an average of tenfold cross validation*.* It is not stated if any feature selection was performed or how missing data were handled. Interestingly, patients were excluded if they did not live at home, and this was one of the important features for predicting mortality in our study. It also sheds light on the impact of different organizations across countries and health care systems. In our study approximately 30% lived in a nursing home compared to approximately 5% in the US study and this is likely to cause issues concerning generalizability with models that use this type of compound parameters as it seems that different circumstances in the general health and socioeconomic status of the individual will lead to the different living arrangements across countries and regions.

Differences in methodology makes comparisons to our study difficult and the intended use of systems that include only easily available preoperative parameters and systems that include large numbers of pre- and post-operative parameters are inherently different.

One of the important parameters in our prediction models was pre-fracture residency. Several previous articles have indicated the importance of this parameter for survival in hip fracture populations. There are several possible causes for this association as it is linked to many other important parameters such as walking status, dementia and comorbidities.

An association between walking status and mortality after hip fracture has been found previously and it is also a constituent in several prediction systems in this field [8, 36, 37]. It seems likely that walking ability in hip fracture patients indicate a less frail patient and, in most cases, it is also a prerequisite for not living in a nursing home. Cognitive frailty is another constituent that could be a factor in the parameter pre-fracture residence as these patients are less likely to live independently.

In a study of 116,111 hip fracture patients in Sweden, shorter length of hospital stay was associated with a higher 30 day mortality [38]. The patients in the early discharge group were also more likely to have dementia. One possible explanation for the association between early discharge and dementia could be that these patients were residents of nursing homes and were discharged early for further care at that institution. Unfortunately, there was no data in this study regarding to what type of living situation patients were discharged.

Overall, the association between pre-fracture residence and mortality might be a representation of the general condition of frail patients and as such represent several parameters previously known to be associated with mortality such as age, mental status, comorbidity and walking status.

While the introduction of geriatric care did not end up as an important predictor in our data, several previous studies have shown that interventions such as dedicated orthogeriatric care perioperatively can decrease postoperative mortality. In a study from the Netherlands, 1-year mortality was decreased from 35 to 23% [39] and several other studies have shown similar results [23, 40]. As these measurements are done on cohorts of “hip fracture patients” as a group, the effect on the subgroup of patients at high risk is likely much higher than that reported. An accurate means of risk stratification could be used to identify which patients might benefit from more intensive monitoring and care in an effort to decrease postoperative mortality and optimize use of healthcare resources.

One of the predictors featured only for 6 and 12 month survival was BMI. Several previous studies have shown an association between low BMI, poor nutritional status and increased mortality in hip fracture patients [41–43]. Evaluation and correction of malnutrition within this timeframe could be interesting to evaluate further.

The presence of cardiac medications/vitamin k-antagonists in the medical history as a parameter in all the prediction models most likely reflects the increased risk of mortality induced by heart conditions as a co-morbidity rather than a risk induced from the medications in their own right. However, it could be of interest to follow patients with this type of cardiac medication and a higher risk of mortality to evaluate if their cardiac status remained unchanged or if the presence of this parameter as a predictor indicates deterioration of their preexisting cardiac morbidity after surgery and perioperative ordeals such as dehydration, decreased mobility, rehabilitation and administered opiates.

Application of machine learning techniques in the emerging age of “big data” in health care has many interesting opportunities and is being used effectively in many other fields already. Hopefully these techniques can provide medical research and clinical decision-making with many new tools. However, while similar methods might be used in areas such as personalized advertising and insurance it will be up to the medical community to ensure that prediction models created by patient data and intended for use in a medical setting is used for the patients’ best medical interest and not commercial purposes. Furthermore, ethical considerations need to be made concerning the intended use and how do distribute results at an early stage even in a medical setting. Identifying patients at risk can be used as an argument both for limiting and optimizing treatment depending on the setting, and decisions about how to handle predictions with a negative outlook and how this information is recorded and communicated during hospital stay and aftercare need to be made so that the overall treatment of patients is not affected negatively.

This study has several limitations. Estimations are based on a population from one single University Hospital in Copenhagen, Denmark, and our study population might not be comparable to other populations. Furthermore, there is always a risk of overfitting predictive models to the study population that they were trained on.

The data are about 10 years old but several large studies have shown that mortality rates after hip fracture have remained stable during the last centuries so for the purpose of creating a predictive model it was considered that this data would not be outdated [44, 45].

Some of these limitations will be investigated in future prospective studies with external validation on other populations that are currently being planned.

There is a risk that the use of pre-fracture residence might not translate well to other populations because of regional differences in health care systems so that a different set of criteria will be used to determine which patients are eligible for assisted living in a nursing home or similar institution.

In conclusion, we successfully developed models capable of estimating 1-, 3-, 6- and 12 month mortality after hip fracture surgery with good discrimination and calibration. The models are based on readily available parameters to facilitate ease of use in a clinical setting.

An online tool based on the XGB models has been developed for educational purposes and is freely available at: (https://hipfx.shinyapps.io/hipfx/).

## Appendix

See Table

**Table 3:** Stratified by 1 year survival

	No	Yes	*p*	NA
*n*	378	808		
Admitted from (%)			< 0.001	
Assisted living	18 (4.8)	52 (6.4)		
Hospice	2 (0.5)	0 (0.0)		
Hospital	7 (1.9)	11 (1.4)		
Nursing home	155 (41.0)	126 (15.6)		
Own home	180 (47.6)	603 (74.6)		
Rehab	16 (4.2)	16 (2.0)		
Permanent/ registered residence (%)			< 0.001	
Homeless	0 (0.0)	1 (0.1)		
Nursing home	154 (40.7)	129 (16.0)		
Own home	220 (58.2)	675 (83.5)		
NA	4 (1.1)	3 (0.4)		
Anesthesia (%)			0.216	
Block	8 (2.1)	15 (1.9)		
Block + epidural	0 (0.0)	1 (0.1)		
Block + epidural, general	0 (0.0)	1 (0.1)		
Block + general	41 (10.8)	61 (7.5)		
Block + sedation	1 (0.3)	2 (0.2)		
Block + spinal	12 (3.2)	32 (4.0)		
block + spinal, general	1 (0.3)	3 (0.4)		
Block + spinal, sedation	0 (0.0)	2 (0.1)		
Epidural	0 (0.0)	5 (0.6)		
Epidural + general	0 (0.0)	1 (0.1)		
General	218 (57.7)	422 (52.1)		
Sedation	2 (0.5)	1 (0.1)		
Spinal	57 (15.1)	185 (22.9)		
Spinal + general	1 (0.3)	6 (0.7)		
Spinal + sedation	2 (0.5)	3 (0.4)		
NA	35 (9.3)	68 (8.4)		
Anasthesia group (%)			0.020	
Combination	60 (15.9)	114 (14.1)		
General	218 (57.7)	421 (52.1)		
Regional	65 (17.2)	205 (25.4)		
NA	35 (9.3)	68 (8.4)		
Fracture type (%)			0.103	
Basocervical	20 (5.3)	42 (5.2)		
Evans i	12 (3.2)	25 (3.1)		
Evans ii	21 (5.6)	75 (9.3)		
Evans iii	25 (6.6)	45 (5.6)		
Evans iv	67 (17.7)	129 (16.0)		
Evans v	35 (9.3)	58 (7.2)		
Garden i–ii	30 (7.9)	105 (13.0)		
Garden iii–iv	133 (35.2)	270 (33.4)		
Intertrochanteric	3 (0.8)	6 (0.7)		
Subtroch—multiple fragments	12 (3.2)	26 (3.2)		
Subtroch—nondisplaced	6 (1.6)	4 (0.5)		
Subtroch—two fragments	12 (3.2)	16 (2.0)		
NA	2 (0.5)	7 (0.9)		
Operation type (%)			< 0.001	
Cannulated screws	34 (9.0)	134 (16.6)		
Cannulated screws, sliding hip screw	0 (0.0)	1 (0.1)		
Dead before operation	5 (1.3)	0 (0.0)		
Hemiarthroplasty	124 (32.8)	220 (27.2)		
Intramedullary nail	153 (40.5)	292 (36.1)		
Resection arthroplasty	2 (0.5)	2 (0.1)		
Sliding hip screw	46 (12.2)	131 (16.2)		
Sliding hip screw, cannulated screws	4 (1.1)	9 (1.1)		
THA	1 (0.3)	10 (1.2)		
NA	9 (2.4)	9 (1.1)		
Operation category (%)			0.053	
Arthroplasty	125 (33.1)	230 (28.5)		
Internal fixation	242 (64.0)	568 (70.3)		
Resection arthroplasty	2 (0.5)	1 (0.1)		
NA	9 (2.4)	9 (1.1)		
New mobility score total (%)			< 0.001	
0	15 (4.0)	10 (1.2)		
1	8 (2.1)	6 (0.7)		
2	60 (15.9)	67 (8.3)		
3	29 (7.7)	49 (6.1)		
4	60 (15.9)	83 (10.3)		
5	12 (3.2)	46 (5.7)		
6	54 (14.3)	116 (14.4)		
7	11 (2.9)	50 (6.2)		
8	1 (0.3)	15 (1.9)		
9	83 (22.0)	315 (39.0)		
NA	45 (11.9)	51 (6.3)		
ASA classification (%)			< 0.001	
1	7 (1.9)	68 (8.4)		
2	134 (35.4)	435 (53.8)		
3	194 (51.3)	266 (32.9)		
4	20 (5.3)	15 (1.9)		
NA	23 (6.1)	24 (3.0)		
BMI (mean (SD))	22.05 (3.47)	22.88 (4.15)	0.001	42
Hemoglobine, mmol/L (mean (SD))	7.36 (1.03)	7.74 (1.05)	< 0.001	
Potassium, mmol/L (mean (SD))	4.02 (0.60)	3.86 (0.48)	< 0.001	3
Sodium, mmol/L (mean (SD))	137.70 (4.72)	137.68 (4.44)	0.950	
Creatinine, μmol/L (mean (SD))	103.84 (74.09)	77.71 (36.54)	< 0.001	1
Calcium, mg/L (mean (SD))	2.26 (0.17)	2.26 (0.13)	0.685	37
Albumine, g/L (mean (SD))	36.49 (5.30)	38.69 (4.27)	< 0.001	11
Glucose, mmol/L (mean (SD))	6.80 (1.99)	6.64 (2.12)	0.228	22
Age (mean (SD))	86.10 (8.38)	81.33 (9.24)	< 0.001	
Sex = male (%)	104 (27.5)	202 (25.0)	0.395	
Leg = right (%)			0.773	1
	181 (47.9)	381 (47.2)		
NSAID = YES (%)	17 (4.5)	53 (6.6)	0.203	
Opioid = YES (%)	99 (26.2)	167 (20.7)	0.040	
Statin = YES (%)	57 (15.1)	155 (19.2)	0.102	
Paracetamol = YES (%)	184 (48.7)	243 (30.1)	< 0.001	
Diuretics = YES (%)	189 (50.0)	289 (35.8)	< 0.001	
Bisfosfonates = YES (%)	23 (6.1)	79 (9.8)	0.045	
Betablockers = YES (%)	75 (19.8)	112 (13.9)	0.011	
Ca channel blockers = YES (%)	79 (20.9)	136 (16.8)	0.107	
ACE inhibitors = YES (%)	62 (16.4)	125 (15.5)	0.745	
ATII inhibitors = YES (%)	17 (4.5)	46 (5.7)	0.474	
Benzodiazepines = YES (%)	54 (14.3)	73 (9.0)	0.009	
Antidepressants = YES (%)	108 (28.6)	173 (21.4)	0.009	
Dementia medication = YES (%)	23 (6.1)	35 (4.3)	0.246	
Calcium/ D3 = YES (%)	116 (30.7)	212 (26.2)	0.127	
Laxatives = YES (%)	106 (28.0)	131 (16.2)	< 0.001	
Methoclopramides = YES (%)	20 (5.3)	25 (3.1)	0.093	
PPI = YES (%)	103 (27.2)	165 (20.4)	0.011	
Antacid magnesium = YES (%)	40 (10.6)	60 (7.4)	0.087	
Antiparkinson medication = YES (%)	3 (0.8)	19 (2.4)	0.105	
Antithrombotics = YES (%)	170 (45.0)	309 (38.2)	0.033	
Heparin = YES (%)	6 (1.6)	6 (0.7)	0.297	
Thrombocyte inhibitors = YES (%)	143 (37.8)	284 (35.1)	0.406	
Vitamin K antagonists = YES (%)	24 (6.3)	28 (3.5)	0.035	
Systemic steroids = YES (%)	16 (4.2)	28 (3.5)	0.626	
Adrenergic inhalations = YES (%)	40 (10.6)	59 (7.3)	0.073	
Anticholinergic inhalations = YES (%)	18 (4.8)	24 (3.0)	0.165	
Steroid inhalations = YES (%)	6 (1.6)	13 (1.6)	1.000	
Digoxine = YES (%)	44 (11.6)	38 (4.7)	< 0.001	
Antipsychotics = YES (%)	35 (9.3)	58 (7.2)	0.260	
Thyroid hormone = YES (%)	16 (4.2)	52 (6.4)	0.166	
Antithyroid hormone = YES (%)	7 (1.9)	8 (1.0)	0.338	
Insulin = YES (%)	14 (3.7)	30 (3.7)	1.000	
Antidiabetics excl insulin = YES (%)	16 (4.2)	40 (5.0)	0.692	
Antibiotics = YES (%)	29 (7.7)	27 (3.3)	0.002	
Organic nitrates = YES (%)	30 (7.9)	32 (4.0)	0.006	
Orthogeriatric ward = YES (%)	279 (73.8)	619 (76.6)	0.330	
BMI_catg (%)			0.001	
High	60 (15.9)	207 (25.6)		
Low	80 (21.2)	148 (18.3)		
Mid	238 (63.0)	453 (56.1)		
Cardiac medication = YES (%)	236 (62.4)	358 (44.3)	< 0.001	

^3^.
